# Concerted Action of Sphingomyelinase and Non-Hemolytic Enterotoxin in Pathogenic *Bacillus cereus*

**DOI:** 10.1371/journal.pone.0061404

**Published:** 2013-04-16

**Authors:** Viktoria M. Doll, Monika Ehling-Schulz, Roger Vogelmann

**Affiliations:** 1 Abteilung Mikrobiologie, Zentralinstitut für Ernährungs- und Lebensmittelforschung ZIEL, Technische Universität München, Freising Weihenstephan, Germany; 2 Functional Microbiology, Department of Pathobiology, University of Veterinary Medicine Vienna, Vienna, Austria; 3 Second Department of Internal Medicine, Universitätsmedizin Mannheim, University Heidelberg, Mannheim, Germany; University of Illinois at Chicago College of Medicine, United States of America

## Abstract

*Bacillus cereus* causes food poisoning and serious non-gastrointestinal-tract infections. Non-hemolytic enterotoxin (Nhe), which is present in most *B. cereus* strains, is considered to be one of the main virulence factors. However, a *B. cereus* Δ*nheBC* mutant strain lacking Nhe is still cytotoxic to intestinal epithelial cells. In a screen for additional cytotoxic factors using an *in vitro* model for polarized colon epithelial cells we identified *B. cereus* sphingomyelinase (SMase) as a strong inducer of epithelial cell death. Using single and double deletion mutants of *sph*, the gene encoding for SMase, and *nheBC* in *B. cereus* we demonstrated that SMase is an important factor for *B. cereus* cytotoxicity *in vitro* and pathogenicity *in vivo.* SMase substantially complemented Nhe induced cytotoxicity *in vitro*. In addition, SMase but not Nhe contributed significantly to the mortality rate of larvae *in vivo* in the insect model *Galleria mellonella*. Our study suggests that the role of *B. cereus* SMase as a secreted virulence factor for *in vivo* pathogenesis has been underestimated and that Nhe and SMase complement each other significantly to cause full *B. cereus* virulence hence disease formation.

## Introduction

*Bacillus cereus* is a spore forming Gram-positive bacterium that is found in natural habitats like soil and plants. Their spores can easily enter the food processing chain due to heat and dryness resistance. *B. cereus* is commonly accepted as a food borne pathogen causing mostly mild food borne gastroenteritis. Nevertheless, fatal outbreaks of *B. cereus* food poisoning [1], [2] and local and systemic non-gastrointestinal-tract infections (e.g. endophthalmitis, pneumonia, sepsis) in humans have been reported [3]. Two types of gastrointestinal diseases can be distinguished. Ingestion of the emetic toxin cereulide, a cyclic dodecadepsipeptide, causes nausea and vomiting, whereas the diarrheal syndrome has been associated mainly with the enterotoxins cytotoxin K (CytK), hemolysin BL (Hbl) and non-hemolytic enterotoxin (Nhe) [4], [5].

In contrast to food borne diseases, far less is known about non-gastrointestinal-tract infections resulting in severe septicemias. In recent years *B. cereus* has been increasingly recognized as an opportunistic human pathogen [6], [7]. Since *B. cereus* is also an insect pathogen, insect models have been widely used to assess *B. cereus* virulence *in vivo*. Especially lepidopteran larvae of the great wax moth *Galleria mellonella* have emerged as a popular insect model for evaluating overall microbial pathogenicity *in vivo* [8]–[11]. Comparable virulence data have been obtained in *G. mellonella* and the obviously very different mouse model [12]. *B. cereus* proteins like hemolysin II and the immune inhibitor A family have been associated with virulence *in vivo* in the insect host [13], [14], whereas major *B. cereus* enterotoxins such as Nhe, Hbl and CytK were poorly examined.

Expression of the secreted enterotoxins Nhe, Hbl and CytK is controlled by the PlcR regulon via a quorum sensing system (for review see [15]). Deletion of *plcR* abolishes *in vitro* cytotoxicity and reduces significantly *in vivo* pathogenicity in mice and insects [12]. PlcR regulates more than 40 genes including degradative enzymes, proteases and phospholipases such as phosphatidylinositol-specific phospholipase C (PI-PLC), phosphatidylcholine-specific phospholipase C (PC-PLC) and sphingomyelinase (SMase) [15]. Synergistic interaction of PC-PLC and SMase for complete lysis of human erythrocytes has been demonstrated and therefore, both enzymes have been proposed to form a cytolytic unit, named cereolysin AB [16]. *B. cereus* sphingomyelinase is structurally related to *Staphylococcus aureus* beta toxin, *Clostridium perfringens* alpha toxin and the sphingomyelinase of the intracellular pathogen *Listeria ivanovii* [16], [17]. Sphingomyelinase is a metal ion-dependent phospholipase that hydrolyzes sphingomyelin in the plasma membrane to phosphocholine and ceramide. Recently gangliosides, especially NeuAcα2-3Galβ1-4Glcβ1-1ceramide (GM3), have been shown as a direct binding site for SMase. The sialic acid of GM3 serves as a cell receptor for binding the β-hairpin region of SMase [18].

Oda *et al.* demonstrated that *B. cereus* SMase is an important virulence factor for septicemia. *B. cereus* strains displaying SMase activity are able to grow *in vivo* in mice after intraperitoneal injection in contrast to strains without SMase activity [19]. Earlier work by Beecher *et al.* suggested that SMase acts in connection with the hemolytic enterotoxin complex Hbl [20]. However, *hbl* genes are restricted to certain strains whereas the *nhe* operon encoding the so called non-hemolytic enterotoxin complex Nhe has been found in all strains tested so far [21]–[23]. Nhe is a pore-forming toxin that requires the expression of its individual enterotoxin components Nhe A, B and C in a certain ratio (10∶10:1) [24] and specific binding order for full *in vitro* cytotoxicity [25]. At least two or three Nhe components are necessary for the formation of functional membrane pores depending on the target cell type [26]. After binding of NheC and NheB to the cell surface a conformational change might occur that allows subsequent binding of NheA, resulting in cell lysis [25]. It is assumed that Nhe is the major enterotoxin playing a key role in *B. cereus* induced diarrhea [27] but a synergistic interaction between SMase and Nhe in *B. cereus* virulence has not been studied so far. Therefore, we examined the interaction of Nhe and SMase for *in vitro* cytotoxicity and *in vivo* pathogenicity. In this work, we provide evidence that *B. cereus* SMase enhances Nhe cytotoxicity in an *in vitro* model for polarized colon epithelial cells. SMase *in vitro* cytotoxicity depends on the presence of Nhe. Furthermore, in the insect model *Galleria mellonella* deletion of the SMase gene *sph* but not *nheBC* significantly reduced larvae mortality. Inactivation of both gene loci indicated that SMase and Nhe cooperate in *B. cereus* pathogenicity *in vivo.*

## Materials and Methods

### Bacterial Strains, Plasmids and Culture Conditions

*Bacillus cereus* NVH 0075-95 [28], its isogenic mutant strains and *E. coli* strain were routinely grown in Luria-Bertani (LB) broth or on LB agar plates at 30°C or 37°C, respectively. For cytotoxicity screening bacterial supernatants were collected from 100 ml cultures in LB broth (37°C, 150 rpm) 8 h after inoculation with 10^3^ colony-forming units (CFU)/ml from a 16-hour pre-culture (corresponding to early stationary phase). When required, bacterial supernatants were concentrated up to 30-fold at 4°C using Amicon® centrifugal filter devices (10 kDa, Millipore). Supernatants were stored at –80°C. Unless stated otherwise, cultures were grown in 200 ml flasks and optical density at 600 nm (OD_600_) was recorded. Samples exceeding an OD_600_ of 1 were diluted 1∶10 and extrapolated.

Where appropriate a single or a combination of the following antibiotics was added to the media: ampicillin (100 µg/ml), kanamycin (50 µg/ml), spectinomycin (100 µg/ml), polymyxin B (50 µg/ml), erythromycin (3 µg/ml), chloramphenicol (5 µg/ml) or tetracycline (10 µg/ml). All bacterial strains and plasmids constructed and used in this study are listed in supplemental Table S1 and S2.

For phenotypic characterization of *B. cereus* wild type (WT) and mutant strains MYP agar (mannitol egg yolk polymyxin; Oxoid) and Columbia agar (containing 5% sheep blood, Oxoid) was used. Therefore, small amounts of colony material were transferred to the respective agar plate surface using the tip of a needle and incubated at 30°C for 24 h. To test for extracellular cross-complementation of hemolytic activity, *B. cereus* NVH 0075-95 WT, Δ*nheBC,* Δ*sph* and Δ*nheBC*Δ*sph* mutants were stricken out crosswise on sheep blood agar plates as described before [16] similar to diagnostic CAMP test and incubated at 30°C for 24 h.

### General Molecular Methods

For cloning, genomic DNA of *B. cereus* NVH 0075-95 served as a template. DNA isolation, manipulation and transformation in *E. coli* was carried out in accordance with standard protocols [29]. All oligonucleotides used in this study are listed in Table S2. A high-fidelity Platinum® *Taq* DNA polymerase (Invitrogen) was used for DNA amplification and DNA manipulations were verified by sequencing.

### Epithelial Cell Cytotoxicity

*Ptk*6 null epithelial cells (Ptk6) from mouse colonic mucosa were cultured as outlined previously [30]. In cytotoxicity assays, 1×10^6^ Ptk6 cells were seeded onto 6-well plates and grown until confluence. For *in vitro* cytotoxicity tests Ptk6 cell monolayers were washed twice with DPBS and treated with either vegetative cells of various *B. cereus* strains (Table S3) at an MOI of 1 (2×10^6^ bacteria) or sterile bacterial supernatant at 37°C/5% CO_2_ atmosphere. IEC Changes in intestinal epithelial cell (IEC) morphology, cell rounding and % cell detachment was monitored over time using light microscopy.

### Identification of Cytotoxic Proteins Secreted by *B. cereus*

Concentrated bacterial supernatant of *B. cereus* NVH 0075-95 Δ*nheBC* was dialyzed against PBS (pH 7.4) and total protein concentration was determined using a BCA protein assay (Thermo Scientific Pierce). 1.64 mg of secreted proteins was fractionated by gel filtration using a Superdex-75 10/300 GL column in a ÄKTA Purifier system (GE) with a flow rate of 0.2 ml/min. Proteins were detected by their absorbance at 280 nm. Fractions were tested for cytotoxic activity on Ptk6 cell monolayers. After SDS-gel separation, silver stained protein bands were analyzed by LC/MS/MS on a ThermoFisher LTQ Orbitrap XL mass spectrometer by NextGen Sciences, Ann Arbor, USA. Protein identifications were accepted according to NextGen Science’s guidelines (greater than 90.0% probability, at least 2 identified peptides.) Protein probabilities were assigned by the Protein Prophet algorithm [31].

### Construction of *B. cereus* NVH 0075-95 sph Deletion Mutants

*Sphingomyelinase* null mutants and complemented strains were generated as described before [32]. In brief, DNA regions of approximately 1200 bp flanking the *sph* gene were amplified by PCR using primers shown in Table S2, primarily inserted into the cloning vector pCR 2.1 TOPO flanking a chloramphenicol resistance cassette. The construct was cut with *SacI/HindIII* and inserted in the similarly digested conjugative suicide vector pAT113. The resulting vector pAT113Δ*sph*/*cm* was transferred into *B. cereus* NVH 0075-95 WT and Δ*nheBC* via conjugation using the donor strain *E. coli* JM83/pRK24 as described elsewhere [33]. Transconjugants were screened for chloramphenicol resistance and erythromycin sensitivity. To confirm gene deletion and integration of the resistance cassette, immunoblotting, PCR and sequencing using the primer pair sph_Sac_F/sph_Hind_R, Cm_F/Cm_R_Not or InCm2_F/sph_downstream_R were carried out. The *sph* deletion mutants were designated *B. cereus* NVH 0075-95 Δ*sph* and Δ*nheBC*Δ*sph.*

### Complementation of sph Deletion Mutants

To complement *sph* deletion mutants, plasmids containing one of the two putative *plc-sph* operon promoter regions P*plc* and P*sph* directly upstream of the *sph* coding sequence were constructed. Using the primer pair Pplc_for/Pplc_rev a 538 bp region upstream of the *plc* gene (promoter P*plc-sph* of the *plc-sph* operon) was amplified from *B. cereus* NVH 0075-95 genomic DNA. The PCR product was digested with *EcoRI* and *SacI* and was ligated in the similarly digested pAD123 vector containing an additional tetracycline resistance cassette (pAD/tet). In a second PCR reaction the promoterless sph coding sequence was amplified using the primer pair sph_Sac_F/sph_Hind_R. After amplification the PCR product was cloned into the *SacI/HindIII* restriction site of the same plasmid leading to the shuttle-vector pAD/*sph*/P*plc*/tet. As control the *sph* gene including a 499 bp upstream region (putative promoter P*sph* described before [34], [35]) was PCR amplified using the primer pair Psph1_for/sph_Hind_R. After restriction digest the PCR product was inserted into the *EcoRI-HindIII* site of pAD/tet giving rise to a plasmid designated pAD/*sph/*P*sph*/tet. Both resulting plasmids were propagated in non-methylating *E. coli* INV110 and introduced into the *B. cereus* NVH 0075-95 *sph* single and triple mutant strains by electroporation as described previously [36]. PCR analysis with Tet^r^-specific primers confirmed the introduction of the plasmids into the generated mutant strains *B. cereus* NVH 0075-95 Δ*sph* comP*plc*, Δ*sph* comP*sph*, Δ*nheBC*Δ*sph* comP*plc* and Δ*nheBC*Δ*sph* comP*sph.* Expression of SMase was confirmed by immunoblotting and SMase activity assay.

### Flow cytometric Analysis of *B. cereus* Cytotoxicity

For flow cytometric analysis, 1×10^6^ Ptk6 cells were seeded onto 6-well plates and grown until confluence. For the assessment of bacterial cytotoxicity, Ptk6 cell monolayers were washed twice with DPBS, treated with serial dilutions of bacterial supernatant in RPMI 1640. After 4 h at 37°C/5% CO_2_, epithelial cells were washed, trypsinized and stained with Propidium iodide (Biozol) to detect necrotic cells. Samples containing 1–2×10^6^ cells were analyzed for total and dead cell numbers using a Gallios flow cytometer (Beckman Coulter). To decipher the effect of SMase for *B. cereus* virulence in vitro, confluent Ptk6 cells were treated with sublethal dilutions (1∶16) of supernatant of *B. cereus* NVH 0075-95 Δ*sph* or Δ*nhe*BCΔ*sph* supplemented with 0, 0.05, 0.1 and 0.2 U/ml of recombinant SMase (Sigma, S9396). To neutralize Nhe activity in the supernatant of Δ*sph*, a monoclonal anti-NheB antibody (1E11) kindly provided by Richard Dietrich [27] was added to wells (10 µg/well) supplemented with 0, 0.1 and 0.2 U/ml of recombinant SMase. After 4 h incubation at 37°C/5% CO_2_, cells were analyzed by flow cytometry as described above.

### PC-PLC and SMase Activity Assay

For enzyme activity assays supernatants of *B. cereus* wild type, the *nheBC* and *sph* mutants and their complemented derivatives cultivated in LB broth at 37°C were harvested (6500 *g*, 4 min, 4°C) at an OD_600_ of 4 and 7 corresponding to transition and early stationary growth phases, respectively. Using an Amplex® Red Sphingomyelinase Assay Kit (Invitrogen/Molecular Probes) production of sphingomyelinase was determined, while synthesis of phosphatidylcholine-specific phospholipase C was quantified with the EnzChek Direct Phospholipase C Assay Kit (Invitrogen/Molecular Probes) following the manufacturer‘s protocol. PC-PLC activity assay was carried out as described before [37] and sphingomyelinase assay was done in accordance except for the following changes. For SMase activity the reactions were developed for 60 min at 37°C in a light-protected microplate thermo-shaker and fluorescence was measured with a Wallac 1420 Victor^2^ multilabel plate reader (Perkin Elmer) at excitation/emission wavelengths of 530/585 nm. Samples of three independent cultures were examined for their phospholipolytic activities. To account for different protein concentrations of the samples enzyme activity was normalized to protein concentration of the respective supernatant using a Bradford-based protein assay (Roti-Quant, Carl Roth GmbH), resulting in mU per µg of protein.

### Immunoblotting of SMase

Protein samples were prepared from bacterial supernatants of *B. cereus* NVH 0075-95 WT, isogenic mutant and complemented strains. 4 µg of total *B. cereus* protein were separated via 10% SDS/PAGE as described before [38]. After transfer to a PVDF membrane (Roche) *B. cereus* SMase was detected using a rabbit polyclonal anti-BcSMase antibody (1∶1000) kindly provided by Jun Sakurai [39]. An Alkaline-Phosphatase-conjugated goat-anti-rabbit IgG (1∶30000) (dianova) was used as second antibody for chromogenic SMase detection.

### Insect Injection Experiments

*Galleria mellonella* (*G. mellonella*) larvae were purchased from Kerf (Unna, Germany) and kept at 15°C. Thirty last-instar *G. mellonella* larvae per condition, weighting 200–400 mg were kept in groups of 10 larvae per box at 15°C. Injection of 5 µl suspensions of vegetative bacteria was done intrahemocoelically into the base of the last left proleg of the larvae. 1.2–2.2×10^5 ^CFU per larva for *B. cereus* NVH 0075-95 WT, Δ*nheBC*, Δ*sph*, Δ*nhe*BCΔ*sph* and the complementation mutant Δ*nhe*BCΔ*sph* comP*plc* and 7.0×10^5^ CFU per larva for *E. coli* DH10B were injected, respectively. Bacterial cell counts of the injected suspension were determined for all strains by plating serial dilutions of the inocula on appropriate medium. *G. mellonella* survival was determined daily over 7 days at 15°C. Larvae were considered dead if they failed to respond to stimulation with a forceps. All tests were run in duplicates with 30 larvae per experiment and condition and they gave similar results for each experiment.

To determine the time-course of bacterial multiplication within *G. mellonella* larvae, 5 larvae of each condition (different injected bacterial strains) were homogenized individually at time-points indicated in the diagram and serial dilutions of the homogenate were plated on LB agar plates for determination of CFU per larva. Larvae were surface-cleaned with 70% Ethanol prior to homogenizing. Bacterial growth monitoring was done in two independent experiments.

### Software and Statistical Analysis

Mean values and S.E. were calculated from at least three independent experiments. For statistical analysis, 2-tailed Student’s *t*-test was used, where applicable, to determine statistically significant differences as indicated in figure legends (GraphPad Prism, Graph Pad Software). Figures were assembled with Photoshop CS and Illustrator CS (Adobe Systems). Raw data of the insect experiments were analyzed using GraphPad Prism (version 5.0) software and plotted according to Kaplan-Meier. Curves were compared for statistical differences using log-rank analysis, which generates a *P* value testing the null hypothesis that the survival curves are identical. *P* values of 0.05 or less were considered significantly different from the null hypothesis.

## Results

### PlcR-mediated Cell Cytotoxicity in Intestinal Epithelial Cells

In order to analyze the role of cytotoxicity factors in *B. cereus* infection, 14 different *B. cereus* strains, which vary in their known toxin gene profile (Tab. S1), were tested for cell cytotoxicity in polarized intestinal epithelial cells (IECs) *in vitro*. As a cell culture model for polarized IEC we used colon epithelial cells derived from *Ptk6* null mice [30].

**Figure 1:**
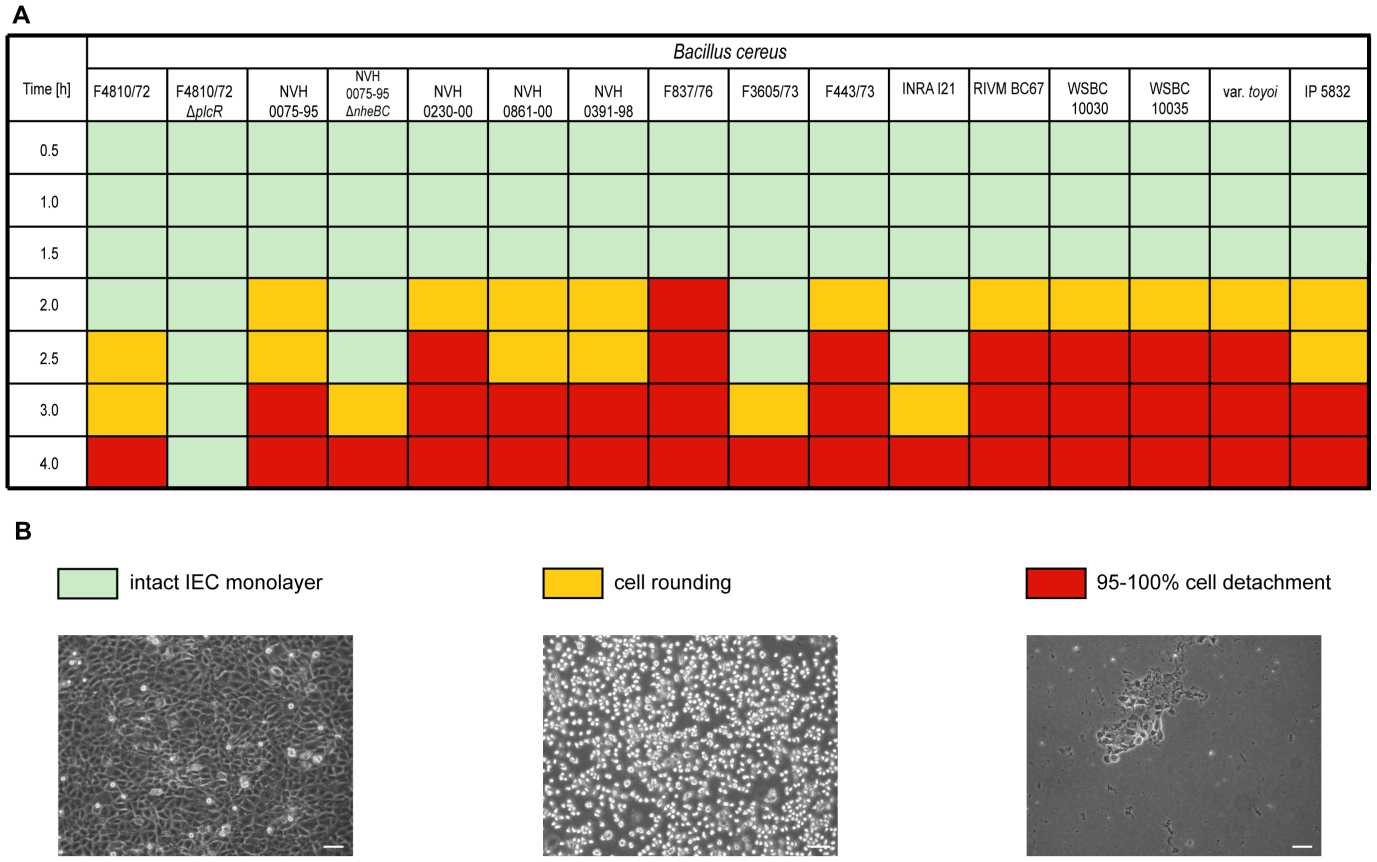
Cytotoxic effects of various *B. cereus* strains on intestinal epithelial cells (IEC). Ptk6 cells were treated with 14 different *B. cereus* strains and two isogenic mutants, morphological changes were monitored over time using light microscopy. **A.** At an MOI of 1 all strains tested caused epithelial cell rounding (yellow) and detachment (red) within 2–4 h after infection except for the *plcR* deletion mutant. Intact monolayer (green). **B.** Representative images of Ptk6 cell monolayers. Bar, 20 µm.

Infection of Ptk6 IEC with various *B. cereus* strains at an MOI of 1 caused rapid cell death with a complete detachment of cells from the tissue culture plate within 4 h (Fig. 1). Although the beginning of cellular rounding and detachment varied between 2 and 4 h there was not a gross difference between strains tested. Even two *B. cereus* strains isolated from two different probiotic formulas used in humans and piglet feeding [40]–[43], *B. cereus* var. *toyoi* and IP5832, revealed similar cytotoxicity compared to *B. cereus* NVH 0391-98 isolated from a food borne outbreak associated with high patient mortality [1] or the *nhe* reference strain *B. cereus* NVH 0075-95 (Fig. 1). Cellular cytotoxicity was mediated by a secreted *B. cereus* factor as rapid rounding and detachment in IEC was induced by bacterial supernatants alone. (Fig. S1). Infection of two different human colonic epithelial cell lines Caco-2 and T84 showed similar results (data not shown).

**Figure 2:**
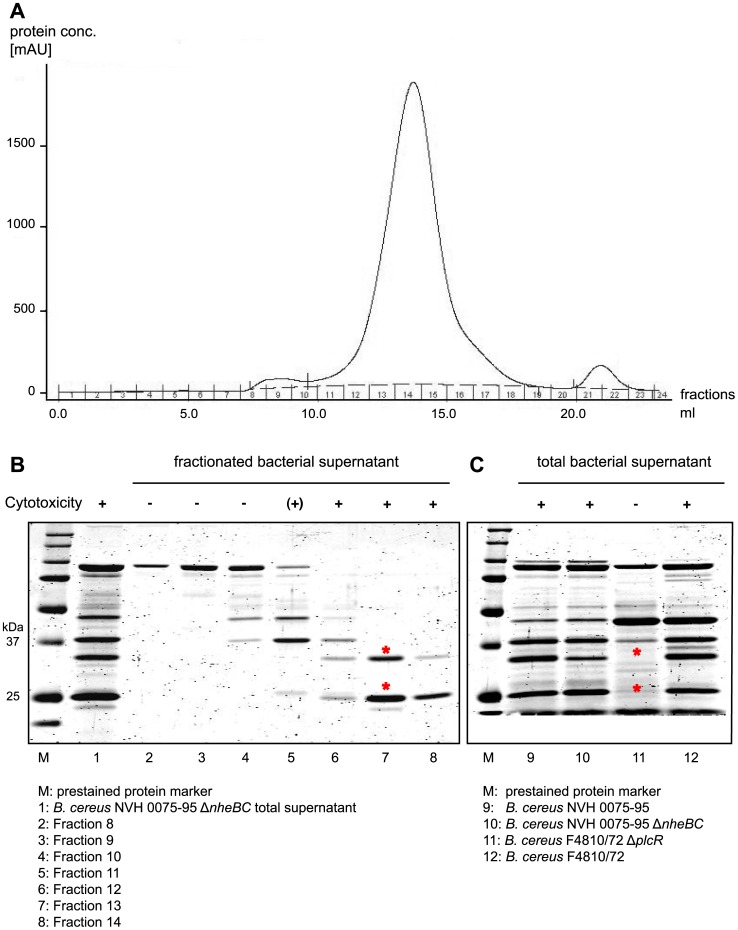
Identification of cytotoxic protein from *B. cereus* supernatant. **A.** Bacterial supernatant of *B. cereus* NVH 0075-95 Δ*nheBC* was separated on a Superdex-75 10/300 GL gel filtration column. Chromatogram of fractionated bacterial proteins is shown (fraction1–24). Protein fractions were tested on Ptk6 cells for cytotoxicity as described in Experimental procedures. Protein fractions obtained from gel filtration were analyzed by SDS-PAGE. **B.** Gel filtration fractions 12–14 transferred cytotoxicity to Ptk6 cells and contained two distinct proteins migrating at 34 kDa and 25 kDa (red asterisks). **C.** Comparing total extracellular proteins of WT and mutant *B. cereus* strains, two potential cytotoxic proteins (red asterisks) were absent in the supernatant of avirulent Δ*plcR* strain.

Common to all *B. cereus* strains tested is the presence of the *nhe* operon genes (Tab. S1). A *B. cereus* NVH 0075-95 *nheBC* mutant (Δ*nheBC;* kindly provided by Simon P. Hardy [44]) as well as its supernatant caused a delay of onset of cellular rounding by 1 h compared to wild type, but IEC still detached completely after 4 h and 6 h, respectively (Fig. 1 and S1). Due to *nheB* truncation and *nheC* deletion *B. cereus* NVH 0075-95 Δ*nheBC* is unable to produce a functional Nhe toxin, since maximal cytotoxic activity of Nhe depends on at least two Nhe components [24]. Cytotoxicity was completely abolished using a *B. cereus* mutant strain lacking the PlcR regulon (*B. cereus* F4872/10 Δ*plcR*) [32]. These data indicate that additional PlcR regulated proteins contribute to IEC cytotoxicity. As *B. cereus* NVH 0075-95 does not express enterotoxins like Hbl or CytK, we screened for other cytotoxic components in the *B. cereus* NVH 0075-95 Δ*nheBC* supernatant using fast protein liquid chromatography (FPLC). Proteins contained in the bacterial supernatant were separated according to their size on a Superdex-75 10/300 GL gel filtration column (Fig. 2A). Fractions 11–18 contained most of the extracellular bacterial proteins. IEC cytotoxicity was confined to three fractions by infecting polarized epithelial monolayers with each of the fractions of the bacterial supernatant of *B. cereus* NVH 0075-95 Δ*nheBC*. Only fractions 12–14 conveyed complete epithelial cell detachment. SDS gel electrophoresis analysis of fractions 8–14, as well as unfractionated supernatant is shown in Fig. 2B and 2C. Comparison of proteins contained in the cytotoxic fractions 12–14 with unfractionated mutant and wild type *B. cereus* supernatants revealed a 34 kDa and a 25 kDa protein band (indicated in Fig. 2B by red asterisk) that were absent in the non-cytotoxic supernatant of *B. cereus* F4810/72 Δ*plcR* mutant (Fig. 2C). Protein identification of the two silver stained protein bands was carried out by NextGen Sciences (AnnArbor, USA) using LC/MS/MS on a ThermoFisher LTQ Orbitrap XL mass spectrometer. Mass spectrometry analysis of the 34 kDa protein identified sphingomyelin phosphodiesterase (sphingomyelinase) of *B. cereus* (NCBI accession number YP_002336808) by two unique peptide sequences, (K)DHANPSFVENK(V) and (K)VQYVFANGCGPDNLSNK(G), and a sequence coverage of 74%. The 25 kDa protein band was identified as hybrid cereolysin AB (NCBI accession number CAA45501). Hybrid cereolysin AB was chosen by Gilmore *et al.* for the functional unit of phospholipase C (5′-terminal region) and sphingomyelinase (3′-terminal region) proposed to work together as the hemolytic determinant hybrid cereolysin AB of *B. cereus* with an expected molecular mass of 67 kDa [34]. The peptides identified by mass spectrometry are unique for the 3′-terminal part of hybrid cereolysin AB, which is identical to the sphingomyelinase (SMase) polypeptide. Therefore, since the smaller protein band at approximately 25 kDa is co-detected with the 34 kDa SMase protein band by immunoblot using anti-BcSMase antibody (data not shown), we propose that the 25 kDa protein might be an isoform or degradation product of sphingomyelinase.

### Construction of *B. cereus* sph Gene Deletion Mutant

**Figure 3:**
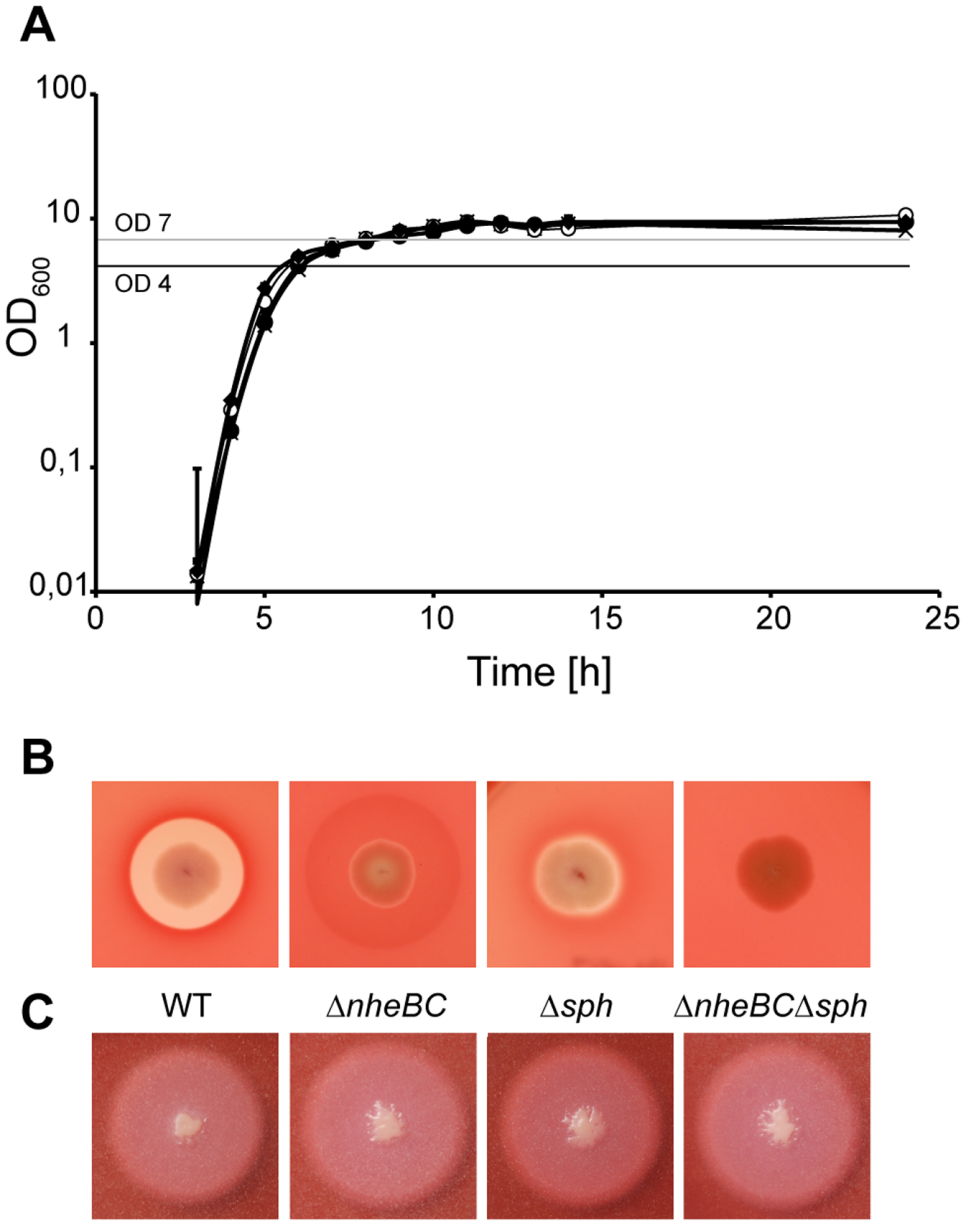
Growth behavior and colony phenotypes of parental and mutant *B. cereus* strains. *B. cereus* NVH 0075-95 (WT, •), isogenic *nheBC (*Δ*nheBC*, ×*), sph (*Δ*sph,* ♦*) and nheBC sph (*Δ*nheBC*Δ*sph,* <$>\raster="rg1"<$>*)* null mutants. Strains were cultivated in LB at 37°C and growth was monitored by measuring optical density at 600 nm (OD_600_) (**A**). Error bars represent standard errors derived from n* = *3 independent experiments. Colony morphology on (**B**) Columbia Blood agar (5% sheep blood, Oxoid) and (**C**) MYP agar (mannitol egg yolk polymyxin agar, Oxoid) was used for specific detection of beta-hemolytic (visible as cleared zones around colonies) and PC-PLC enzyme activity (lecithin precipitation zone), respectively.

In order to address the question to which extent SMase contributes to cellular cytotoxicity *in vitro*, sphingomyelinase null mutants (Δ*sph*) were constructed. Allelic gene replacement of the *sph* ORF by a chloramphenicol resistance cassette resulted in the *B. cereus* strains NVH 0075-95 Δ*sph* and NVH 0075-95 Δ*nheBC*Δ*sph (sph::cm)*. *B. cereus* NVH 0075-95 WT and the isogenic mutants Δ*nheBC*, Δ*sph* and Δ*nheBC*Δ*sph* grew identical in LB medium at 37°C (Fig. 3A). On sheep blood agar, *B. cereus* WT showed strong beta-hemolytic activity indicated as clear zones around colonies (Fig. 3B). The hemolysis zone in the *nhe* mutant was markedly reduced in clearance albeit not in diameter compared to WT, while *sph* deletion abolished most but not all beta-hemolytic activity. The additional *sph* deletion resembled a Δ*plcR*-like hemolytic-negative phenotype in *B. cereus* NVH 0075-95 Δ*nheBC*Δ*sph* suggesting that in Hbl^-^, HlyII^-^ *B. cereus* strains like NVH 0075-95 the hemolytic phenotype is conveyed by Nhe and SMase.

### Sph Null Mutation does not Affect PC-PLC Activity

**Figure 4:**
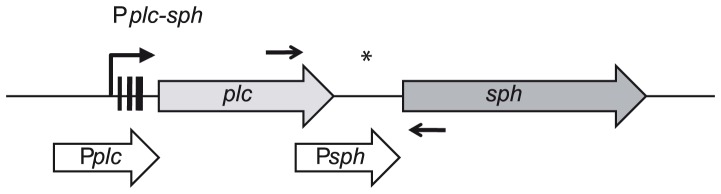
Genomic organization of the chromosomal *plc – sph* gene cluster. The operon comprises phosphatidylcholine-specific phospholipase C (*plc*) and sphingomyelinase (*sph*). P*plc-sph* refers to the putative promoter region of the *plc-sph* operon. Three black bars in the promoter region represent the identified −35, −10 boxes and the ribosome-binding site. P*plc* (white thick arrow) designates a 538-bp fragment spanning the operon promoter region P*plc*-*sph* used for successful complementation, while the inter-genic region is covered by P*sph* (499bp; white thick arrow). The position of inplc_for and insph_rev primer binding for co-transcription analysis are indicated by two small black arrows. *putative promoter region directly upstream of *sph* start codon (P*sph*).

Since *plc* and *sph* are arranged in one operon (cereolysin AB operon, Fig. 4), the 5′ adjacent *plc* gene might have been affected by incorrect double crossover during allelic replacement of *sph*. To test for any polar effects, the mutant strains were cultivated on MYP agar. Typically, phosphatidylcholine-specific phospholipase C (PC-PLC) activity resulted in peripheral zones of egg yolk precipitation on MYP agar (Fig. 3C). This phenotype was not affected by inactivation of *nhe* or *sph*. In order to quantify phosphatidylcholine-specific phospholipase C activity of WT and of isogenic *sph* null strains we determined the lipolytic activity of culture supernatants using a substrate-specific assay for PC-PLC detection. To correct for putative differences in protein concentrations of the strain specific secretomes, enzyme activities were normalized to the protein content of each sample. Supernatant samples were taken from cells at OD_600_ = 4, when PC-PLC activity peaked during growth in LB medium at 37°C (data not shown). With 121.6±25.6 mU/µg protein and 120.5±25.2 mU/µg protein, respectively, phospholipolytic activity of *B. cereus* NVH 0075-95 WT and Δ*nheBC* was almost identical. After *sph* deletion PC-PLC activity was still high for *B. cereus* NVH 0075-95 Δ*sph* (173.1±12.8**mU/µg protein) and Δ*nheBC*Δ*sph* (168.9±28.4**mU/µg protein) and not significantly different from WT (*P* = 0.146) and Δ*nheBC* (*P* = 0.271), respectively.

### Sph is co-transcribed with plc from a Common Promoter

For complementation of *sph* null mutants we sought to reconstitute SMase protein expression under its own *sph* promoter in both *sph* knockout strains. Yamada *et al.* [34] and Pomerantsev *et al.* [35] have described a putative promoter region P*sph* directly upstream of the *sph* start codon, although *sph* and *plc* are known to be arranged in one operon (cereolysin AB operon) probably transcribed from the common promoter P*plc-sph* (Fig. 4).

Because of this discrepancy, we used reverse transcriptase PCR to confirm that the *plc-sph* gene cluster was co-transcribed from the operon promoter region P*plc*-*sph* (data not shown). As expected from co-transcription analysis the putative promoter region P*sph* directly upstream of the *sph* start codon failed to reconstitute SMase expression in *B. cereus* NVH 0075-95 Δ*sph* comP*sph* and Δ*nheBC*Δ*sph* comP*sph* (Fig. S2C).

**Figure 5:**
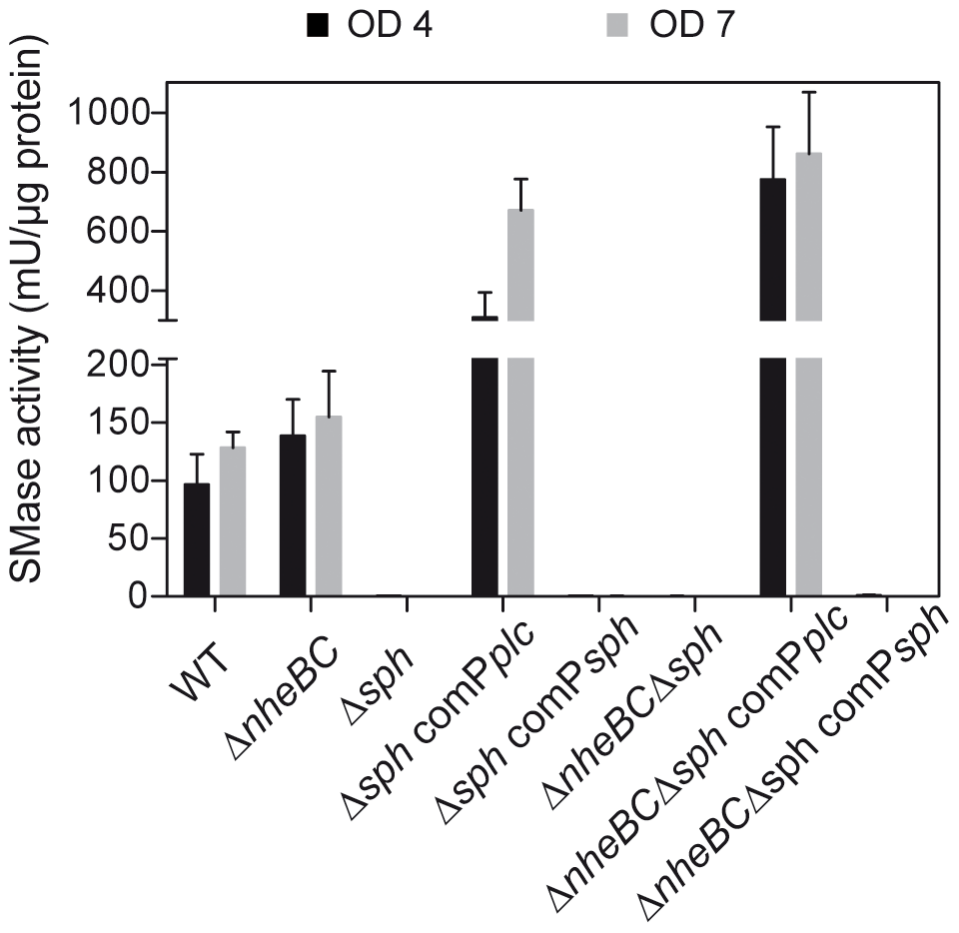
Effect of *sph* deletion and complementation *in trans* on synthesis of SMase enzyme by *B. cereus* NHV 0075-95 and deduced mutant strains. SMase activity was normalized to the protein concentration of prepared supernatant samples. *Sph* deletion completely abolished SMase activity, which could be restored by *in trans* expression of sph driven from the operon promoter region P*plc* at different growth phases; OD_600_ = 4, black bar and OD_600_ = 7, grey bar. Data represent mean values ± SEM (n ≥3).

Therefore, *in trans* complementation of *sph* null mutants was achieved by introducing the plasmid pAD/*sph*/P*plc*/tet driving *sph* expression from the operon promoter P*plc-sph*. SMase expression in complemented strains Δ*sph* comP*plc* and Δ*nheBC*Δ*sph* comP*plc* was confirmed by immunoblotting (Fig. S2C). Re-introduction of *sph* restored beta-hemolytic enzyme activity, while lipolytic activity was not affected (Fig. S2A and S2B). SMase enzyme activity was significantly enhanced in complemented strains restoring wild type activity by 321–558% due to the multicopy nature of the complementation vector pAD (Fig. 5).

### SMase adds to Nhe Induced *B. cereus* Cytotoxicity in vitro

**Figure 6:**
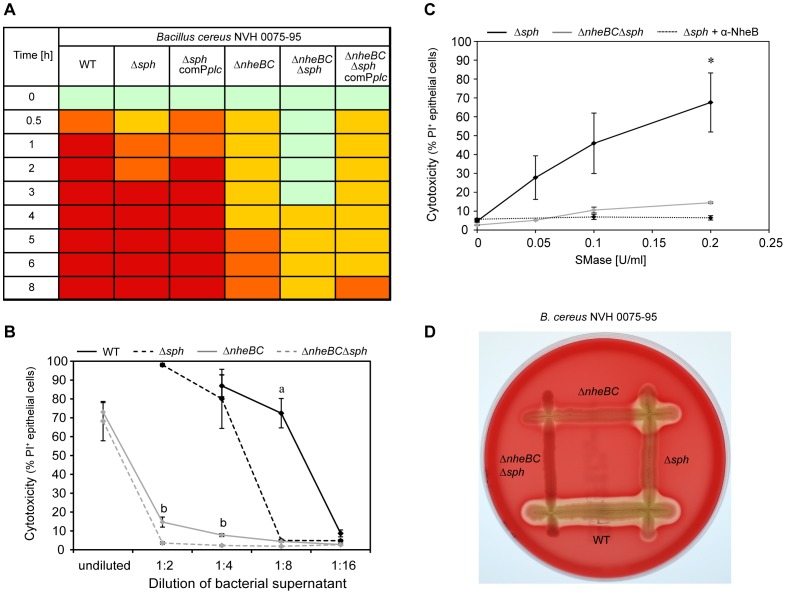
*Sph* deletion effected *B. cereus* virulence *in vitro*. **A**. Cytotoxic effect of sterile *B. cereus* supernatants (1∶4 diluted) on IEC. Intact monolayer (green), cell rounding ≤50% (yellow), cell rounding >50% (orange) and 95–100% cell detachment (red) are indicated. **B**. Cytotoxic effects of *B. cereus* supernatant on IEC were analyzed using flow cytometry. Ptk6 cells were treated with various dilutions of bacterial supernatant of *B. cereus* NVH 0075-95 WT (black line), Δ*nheBC* mutant (grey line), Δ*sph* mutant (black dashed line) and Δ*nheBC*Δ*sph* mutant (grey dashed line). Samples were stained with Propidium iodide (PI) for dead epithelial cells and cytotoxicity is expressed in % of PI positive cells as determined by flow cytometric analysis. Cytotoxicity of the Δ*sph* mutant was strongly reduced at a dilution of 1∶8 compared to WT (a, *P*<0.05). *Sph* deletion in addition to Nhe inactivation significantly reduced cytotoxicity compared to Nhe inactivation alone (b, *P*<0.05). Data plotted represent mean values ± SEM (n = 3). **C**. Cooperative cytotoxic interaction of SMase and Nhe. Addition of various concentrations of recombinant SMase (0.05, 0.1 and 0.2 U/ml) to diluted (1∶16) bacterial supernatants caused significantly higher cytotoxicity against Ptk6 cells when subtoxic Nhe concentrations were present (Δ*sph* supernatant, black line) compared to supernatant without Nhe (Δ*nheBC*Δ*sph* supernatant, grey line) (*P<0.05). Addition of anti-NheB (1E11) antibody (10 µg/well) neutralizes Nhe activity (Δ*sph* supernatant+α-NheB, black dotted line). Cytotoxicity is expressed in % of PI positive cells and data represent mean values ± SEM (n ≥3). **D**. CAMP-like test on sheep blood agar demonstrated complementation of extracellular hemolytic activity between *B. cereus* NVH 0075-95 Δ*sph and* Δ*nheBC*. Beta-hemolytic activity appeared as cleared zone around the colonies.

To elucidate the role of SMase to cellular cytotoxicity *in vitro*, we tested *sph* deletion mutants in our initial screen for colon epithelial cell cytotoxicity based on the morphological characteristics cell rounding and detachment. This analysis revealed that SMase as a single factor contributed little to *B. cereus* cytotoxicity compared to WT (Δ*sph*). However, SMase had a significant effect on IEC cytotoxicity in combination with Nhe (Δ*nheBC*Δ*sph*). Re-expression of SMase in complemented strains Δ*sph* comP*plc* and Δ*nheBC*Δ*sph* comP*plc* reversed the effect (Fig. 6A).

In order to quantify the influence of SMase on cell cytotoxicity, polarized IEC were incubated for 4 h with serial dilutions of bacterial supernatant of *B. cereus* WT and isogenic mutant strains (Fig. 6B). After staining for dead epithelial cells using Propidium iodide (PI), PI positive and total cell numbers were counted using flow cytometry. Mock (LB medium, 1∶2) treated IEC had 1.5%±0.1 PI positive cells. Undiluted supernatants of *B. cereus* NVH 0075-95 WT and *sph* deletion mutant showed high cytotoxicity against IEC resulting in complete detachment and disintegration of epithelial cells rendering them inaccessible for flow cytometric analysis. Wild type cytotoxicity did not drop below 50% before a dilution of 1∶16 (Fig. 6B). Deletion of *sph* significantly reduced *B. cereus* cytotoxicity compared to WT. PI positive cells decreased to 5.0%±0.5 in *B. cereus* NVH 0075-95 Δ*sph* at a 1∶8 dilution compared to 72.4%±7.8 in WT.

Treatment of epithelial cells with supernatant of the *B. cereus* NVH 0075-95 Δ*nheBC* mutant strain resulted in 73.0%±5.0 PI positive cells for undiluted and 14.7%±2.7 for 1∶2 diluted samples. The additional *sph* deletion reduced significantly the number of dead cells to 3.6%±0.6 in *B. cereus* NVH 0075-95 Δ*nheBC*Δ*sph* (*P*<0.05) (Fig. 6B). Our results emphasize the dominant role of the non-hemolytic enterotoxin Nhe for *B. cereus* cytotoxicity against polarized intestinal epithelial cells and at the same time hint to a separate effect of SMase adding to Nhe cytotoxicity.

### SMase Supplements Nhe Cytotoxicity in vitro

To analyze the concerted action of SMase and Nhe for epithelial cell cytotoxicity, we treated polarized IECs with subtoxic dilutions (1∶16) of supernatants of *B. cereus* NVH 0075-95 WT and mutant strains and compared the cytotoxic effect of added purified recombinant SMase. Addition of 0.05, 0.1 and 0.2 U/ml SMase to supernatant of *B. cereus* NVH 0075-95 Δ*nheBC*Δ*sph* consistently increased its cytotoxicity against IECs: 0.2 U/ml SMase increased cell death by 5.4-fold (14.5%±0.5 with compared to 2.7%±0.4 without (*P*<0.0001); Fig. 6C). In contrast when added to supernatant of *B. cereus* NVH 0075-95 Δ*sph*, the addition of 0.05, 0.1 and 0.2 U/ml SMase caused a much greater increase in cytotoxicity in the presence of a non-toxic level of Nhe in the supernatant of Δ*sph*: 0.2 U/ml SMase increased cytotoxicity by 14.4-fold (67.6%±15.6 with compared to 4.7%±0.6 without (*P*<0.001); Fig. 6C).

These results suggest a cooperative mechanism of enterotoxin Nhe and SMase activity in epithelial cell death *in vitro*. To ensure that the increase in cytotoxicity after addition of SMase was due to the remaining Nhe activity in the supernatant of Δ*sph*, we blocked Nhe activity by using a monoclonal anti-NheB antibody (MAb 1E11) [27], which has been shown to block Nhe activity [45], in the supernatant of the *sph* deletion strain. Neutralization of Nhe activity resulted in a strong decrease of SMase cell cytotoxicity in a way similar to the double-knockout Δ*nheBC*Δ*sph* (Fig. 6C) revealing the synergistic effect of Nhe and SMase.

To confirm the synergy of Nhe and SMase proteins a test similar to the classical Christie, Atkins, Munch-Peterson (CAMP) reaction was performed [46]. Neither *B. cereus* single deletion mutants Δ*sph* and Δ*nheBC*, nor the double mutant Δ*nheBC*Δ*sph* showed significant hemolysis of erythrocytes on sheep blood agar (Fig. 6D). However, in the area, where the streaks of *B. cereus* Δ*sph* and *B. cereus* Δ*nheBC* cross, the merging of Nhe (*B. cereus* Δ*sph*) and SMase (*B. cereus* Δ*nheBC*) restored the hemolytic activity comparable to *B. cereus* WT (Fig. 6D).

### Synergistic Interaction of SMase and Nhe for Full *B. cereus* Virulence in vivo

**Figure 7:**
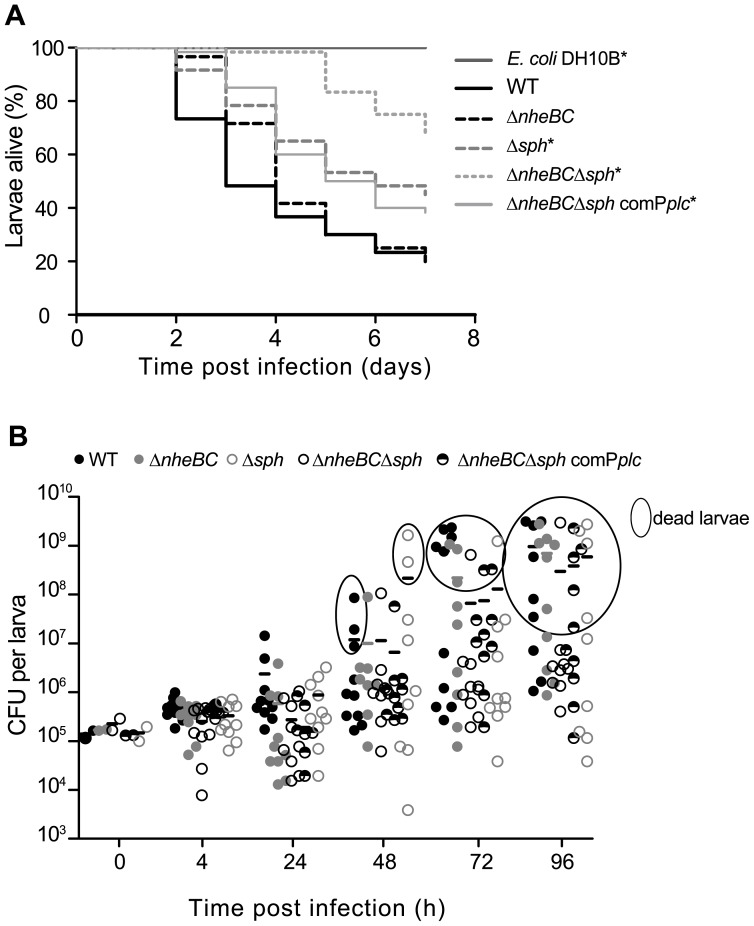
*Sph* deletion strongly reduced the pathogenicity of *B. cereus* NVH 0075-95 in a *Galleria mellonella in vivo* model. **A.** Larvae were infected by intrahemocoelic injection of 10^5^ CFU of vegetative *B. cereus* NVH 0075-95 (WT), the isogenic *nheBC* mutant (Δ*nheBC*), the isogenic *sph* null mutant (Δ*sph*), the *nheBC sph* mutant strain (Δ*nheBC*Δ*sph*) or its complemented strain (Δ*nheBC*Δ*sph* comP*plc*) as described in Materials and Methods. Larvae infected with the non-insecticidal *E. coli* strain DH10B served as control group. *G. mellonella* survival data are plotted as Kaplan-Meier plots. Data are retrieved from two independent infection experiments with a total of 60 larvae per condition. Both experiments showed very similar results. Statistical significance was determined using log-rank analysis. An asterisk indicates treatment groups with a survival distribution function statistically different from *B. cereus* WT (*P*<0.001). **B.** Survival and multiplication of *B. cereus* WT and isogenic mutant strains in *G. mellonella* after intrahemocoelic injection of 10^5^ vegetative cells. Bacterial growth in two independent infection assays was monitored at indicated time points after infection (t* = *0) by counting individual homogenates of five larvae per condition. CFUs recovered from dead larvae are indicated as encircled data points. Paired Student’s *t*-test was used to determine statistical differences between bacterial cell counts of the five treatment groups. In living or dead *Galleria* larvae cell counts of the *sph* mutant strains did not differ significantly from WT and Δ*nheBC* mutant.

To assess the contribution of SMase and Nhe to *B. cereus* pathogenicity *in vivo*, *Galleria mellonella* larvae were used as a model system for bacterial infection. After intrahemocoelic injection of *B. cereus* NVH 0075-95 WT, isogenic mutant strains and *E. coli* DH10B as mock control larvae survival was monitored over seven days (Fig. 7A). All larvae injected with mock control survived the monitoring period. Following injection of *B. cereus* NVH 0075-95 WT mortality rate of *G. mellonella* larvae was >50% after 3 and >75% after 7 days (Fig. 7A). Surprisingly, survival of *B. cereus* NVH 0075-95 Δ*nheBC* injected larvae was not different to WT suggesting that Nhe contributes little to larvae mortality. Deletion of *sph* alone (Δ*sph*) resulted in a significant delay of *G. mellonella* mortality to 22.7%±5.3 after 3 days and 55.0%±6.4 after 7 days. Additional *nheBC* inactivation (Δ*nheBC*Δ*sph*) reduced larvae death further to 1.7%±1.6 dead larvae after 3 days and 31.7%±6.0 after 7 days. Re-expression of SMase in the complemented strain Δ*nheBC*Δ*sph* comP*plc* restored most but not all *in vivo* pathogenicity.

To test whether differences in larvae mortality were attributed to a defect in bacterial growth *in vivo*, the bacterial load in infected larvae was quantified over time. Increasing colony-forming units (CFU) were counted 4–24 h post injection for all strains, suggesting that WT as well as mutant strains are capable of growing in the hemocoel (Fig. 7B). Bacterial load in living larvae increased up to 10^7^ CFU. After larvae death bacterial growth was further enhanced up to 10^9^ CFU (data points encircled, Fig. 7B). We could not detect statistical differences between colony-forming units of WT or mutant strains within living (*P*>0.05) or dead larvae (*P*>0.05) indicating that in our study reduced pathogenicity associated with *sph* deletion was not caused by differences in growth *in vivo*.

Our study shows that *B. cereus* SMase is an important virulence factor for *in vivo* pathogenesis in non-gastrointestinal-tract infections. Furthermore, it demonstrates a cooperative interaction of the major enterotoxin Nhe and phospholipase SMase in *B. cereus* virulence.

## Discussion

*B. cereus* infections in humans are usually mild causing brief episodes of gastrointestinal symptoms and are probably highly underreported [4]. Considering the strong cytotoxic effect on eukaryotic cells in cell culture experiments *in vitro* it is fortunate that the bacteria must lack certain qualities that e.g. their close relative *B. anthracis* seem to possess. Even *B. cereus* strains isolated from probiotic formulas are cytotoxic to eukaryotic cells *in vitro* at a very low MOI of 1, whereas usually high doses of 10^5^–10^8^ enterotoxigenic *B. cereus* cells or spores are required for food poisoning [4], [47]. Once the protective barrier of the mucosa or dermis is overcome, *B. cereus* causes serious and often-fatal non-gastrointestinal-tract infections characterized by extensive tissue destruction [3]. Although *B. cereus* infections in the past have been rarely detected as the causative agent of infectious diseases, recent events demonstrate that new highly virulent pathogens can emerge due to the plasticity of bacterial genomes causing outbreaks with serious outcomes for affected patients [48].

Much of *B. cereus* virulence is mediated by secreted enterotoxins. Three cytotoxins, CytK, Hbl and Nhe, are currently considered to be responsible for *B. cereus* diarrheal disease but of those three only Nhe is present in most *B. cereus* strains currently known [4]. In contrast, enterotoxin genes *hbl* and *cytK* are often absent even from *B. cereus* strains isolated from disease outbreaks, which argues against a prominent role of these two toxins in disease formation [22], [23]. Nhe is considered to be the major factor in *B. cereus* diarrheal disease, because *in vitro B. cereus* cytotoxicity correlates well with Nhe concentration in the bacterial supernatant [49]. Neutralizing antibodies against Nhe abolished almost completely cytotoxicity and a *B. cereus nheBC* mutant NVH 0075-95 lost all cytotoxicity in cell culture based assays [27], [44], [49].

In our study we used the previously described *B. cereus* Nhe mutant strain NVH 0075-95 Δ*nheBC* [44] to identify *B. cereus* sphingomyelinase as an additional virulence factor. Incubation of polarized colon epithelial cells (Ptk6) with the supernatant of the Δ*nheBC* mutant strain for 4 h resulted in nearly 90% of dead cells revealing that other secreted cytotoxins must contribute to epithelial cell cytotoxicity as well. A major difference to the previous study, in which the lack of Nhe in *B. cereus* NVH 0075-95 (Δ*nheBC*) abolished the entire cytotoxicity of the wild type strain, was time of incubation [44]. Fagerlund *et al.* described that the uptake of Propidium iodide into Vero and Caco-2 cells as an indicator for cell death was abolished in the absence of Nhe in the first 15–30 minutes after adding bacterial supernatant [44]. Our observation that the onset of cell rounding and detachment was delayed in *B. cereus* NVH 0075-95 Δ*nheBC* compared to WT supports the finding that Nhe plays a significant role in the early phase of cell cytotoxicity. However, both strains caused cell death and complete detachment of epithelial cells 4 h after adding bacterial supernatants to cells.

Our study confirmed the pivotal role of the PlcR regulon in *B. cereus* mediated cytotoxicity and we identified sphingomyelinase as an important factor contributing to *B. cereus* cytotoxicity. SMase has been described before as a metal ion dependent phospholipase showing hemolytic activity against sheep erythrocytes [50]. The capacity of *B. cereus* to detach host cells *in vitro* correlated positively with the genomic presence of the *sph* gene [51]. Protein homologues of SMase in *S. aureus* (β-toxin) and *Cl. perfringens* (α-toxin) are well-established virulence factors involved in cytotoxicity against host cells [17], [52]. Walev *et al.* demonstrated cytotoxicity of *S. aureus* β-toxin, *Streptomyces sp.* SMase and *B. cereus* SMase against human monocytes [53]. *B. cereus* SMase has been shown to inhibit neurite outgrowth in PC12 cells and to induce membrane damage in host neural cells [54]. However, the role of *B. cereus* SMase as a virulence factor is still under debate, because SMase was nontoxic against Vero cells and in an *in vitro* retinal toxicity model [55], [56]. Differences in cytotoxicity observed between various cell lines may be attributed to variations in phospholipid composition and sphingomyelin content of host cell membranes as seen for red blood cells of different species varying in their sphingomyelin content ranging from 25–53.1% (human – sheep) [20], [57].

In our current study we demonstrate that SMase is a cytotoxic factor *in vitro* and its effect is significantly enhanced in cooperation with Nhe. The significance of SMase to overall *B. cereus* pathogenicity was demonstrated in *sph* mutants in wild type and NVH 0075-95 Δ*nheBC*. *In vitro*, *sph* deletion alone had little effect on epithelial cell cytotoxicity. But it added significantly to Nhe mediated cell cytotoxicity, suggesting a synergistic mode of action for the pore-forming enterotoxin Nhe and SMase. In the absence of the Nhe membrane-binding components B and C (supernatant of Δ*nheBC*Δ*sph*) the addition of recombinant sphingomyelinase resulted in a small but significant increase of epithelial cell cytotoxicity. However, Nhe in the *B. cereus* supernatant (Δ*sph*) enhanced the cytotoxic effect of recombinant SMase to a much higher degree indicating a cooperative interaction of Nhe and SMase via a yet unknown mechanism.

The phenomenon of hemolytic synergy of bacterial phospholipases C and pore-forming toxins has been reported before [20], [57], [58]. Beecher and Wong, 2000, demonstrated interaction of SMase and the hemolytic enterotoxin Hbl for erythrocyte hemolysis [20]. To our knowledge the results presented in this study are the first to demonstrate a complementary effect of the non-hemolytic enterotoxin Nhe and SMase. Nhe *in vitro* cytotoxicity requires a specific binding order of the enterotoxin components Nhe A, B and C [25]. For the formation of functional membrane pores either two or three Nhe components are necessary depending on the target cell type [26]. The secreted sphingomyelinase might adsorb to the epithelial cell surface as demonstrated for erythrocytes [59], thereby modifying membrane structure and fluidity by cleaving the membrane constituent sphingomyelin (SM). This could disrupt membrane integrity followed by moderate levels of epithelial cell lysis. Since the cytotoxic effect of SMase addition was severely enhanced in the presence of Nhe at a non-toxic concentration, Nhe might function as a gatekeeper. This is further supported by the fact that addition of anti-NheB antibody 1E11 reduced SMase induced cytotoxicity in a way similar to the double-knockout mutant Δ*nheBC*Δ*sph*. The antibody 1E11 has been demonstrated to inhibit the association of Nhe A with cell-bound NheB via binding to the C-terminal part of NheB, thereby neutralizing Nhe activity [45].

Once the Nhe complex has formed functional transmembrane pores into the epithelial cell membrane, SMase could enter cells more easily hence reach otherwise inaccessible substrate pools in the inner membrane leaflet. SM hydrolysis could result in cell membrane destabilization as well as cell apoptosis via the ceramide intracellular signaling pathway [60]. Another conceivable mechanism of SMase and Nhe synergism might be that the enzymatic breakdown of membrane sphingomyelin to ceramide by SMase provides access to converted ceramide or other membrane components that can act as binding sites for the active Nhe enterotoxin complex. Such a mechanism has been demonstrated for several bacterial secreted factors such as *S. aureus* β-toxin and the classical *S. agalactiae* CAMP factor or *Propionibacterium acnes* and a host acidic SMase which synergistically enhance lysis of erythrocytes [61], [62]. The CAMP factor of *S. agalactiae* has been similar to *B. cereus* Nhe characterized as an oligomeric pore-forming toxin [63]. Therefore, it is tempting to speculate that *B. cereus* Nhe could be a CAMP-like factor.

The importance of *B. cereus* SMase as a virulence factor is strongly supported by *in vivo* observations in various insects. *B. cereus* SMase caused significant mortality of German cockroaches, cut- and silkworms when applied as a purified enzyme expressed in *B. cereus* or *E. coli* [64], [65]. Insect models in general and larvae of the great wax moth *Galleria mellonella* in particular are widely used for the assessment of bacterial pathogenicity *in vivo,* since the invertebrate immune system functionally resembles the mammalian innate immune response to bacterial infections [8], [10], [14], [66], [67]. In the *G. mellonella* model we demonstrated that *sph* expression is important for the pathogenic phenotype of *B. cereus* NVH 0075-95 suggesting that SMase is a significant virulence factor *in vitro* and *in vivo*, necessary to establish a severe course of infection. These data confirm recent results published by Oda *et al.* demonstrating that SMase is essential for *B. cereus* induced mouse mortality [19]. They showed that SMase mediates bacterial growth of clinical isolates *in vivo* and evasion from the host innate immune system in contrast to environmental *B. cereus* isolates. *B. cereus* strains isolated from soil did not cause lethality in mice due to their lack of SMase, but addition of SMase to *B. cereus* environmental strains injected into the peritoneum reconstituted virulence of clinical isolates causing them to grow *in vivo* [19]. However, in *G. mellonella* SMase may convey mortality in a different way than in mice, because our *sph* deletion mutants did not show differences in growth.

Given the strong role of Nhe for *in vitro* cell cytotoxicity, we were surprised to see that the *B. cereus* NVH 0075-95 Δ*nheBC* mutant strain behaved similar to wild type in *G. mellonella* larvae suggesting that Nhe’s sole contribution to *B. cereus* pathogenicity *in vivo* is maybe less than what is predicted from *in vitro* results. The mortality of *G. mellonella* larvae was significantly reduced in the *B. cereus sph* deletion mutant. The additional inactivation of *nheB*/*nheC* reduced larvae mortality even further, supporting our *in vitro* observation that SMase and Nhe cooperatively determine *B. cereus* virulence.

In summary, our data show that the contribution of SMase to *B. cereus* virulence has been underestimated in the past and our results obtained in cell culture and the *Galleria* model using deletion mutants of *B. cereus* confirm that *B. cereus* SMase contributes in cooperation with Nhe significantly to *in vitro* cytotoxicity and *in vivo* pathogenicity. Therefore, it is tempting to speculate that this is also the case in the human host. Secreted factors like SMase and enterotoxins may interact synergistically to cause tissue destructive effects in human *B. cereus* infections.

## Supporting Information

Figure S1 **Cytotoxic effects of sterile** ***B. cereus*** **supernatants on IEC.** Ptk6 cells were treated with *B. cereus* F4810/72 and NVH 0075-95 WT and isogenic mutant strains. Morphological changes of Ptk6 cells were monitored over time using light microscopy. All diluted supernatants (1∶2) caused immediate epithelial cell rounding and detachment except for the *plcR* deletion mutant. Intact monolayer (green), cell rounding <50% (yellow), cell rounding >50% (orange) and 100% cell detachment (red) are indicated.
(DOC)

Figure S2 **Characterization of** ***sph*** **deletion mutants, complemented and parental** ***B. cereus*** **strains.** **A.** Hemolytic activity of *B. cereus* WT and isogenic mutant strains on Columbia agar (5% sheep blood, Oxoid). **B.** Colony morphology of WT and isogenic mutants on MYP (mannitol egg yolk polymyxin) agar indicating PC-PLC enzyme activity. **C.** Western blot analysis of SMase expression using a polyclonal anti-BcSMase antibody (1∶1000). Cells were grown in LB at 37°C and supernatants were harvested at similar OD_600_. Identical amounts (4 µg) of total protein preparations were separated on a 10% SDS-polyacrylamide gel and transferred to a PVDF membrane. Lanes: 1, *B. cereus* NVH 0075-95 (WT); 2, *nheB* truncation and *nheC* deletion mutant strain of NVH 0075-95 (Δ*nheBC*); 3, *sph* deletion mutant of NVH 0075-95 (Δ*sph*); 4, NVH 0075-95 Δ*sph* comP*sph*, *sph* deletion harboring pAD/*sph*/P*sph*/tet; 5, NVH 0075-95 Δ*sph* comP*plc*, *sph* deletion complemented via pAD/*sph*/P*plc*/tet driving *sph* transcription from the operon promoter region P*plc-sph*; 6, *nheBC* inactivation and *sph* deletion mutant of NVH 0075-95 (Δ*nhe*BCΔ*sph*); 7, NVH 0075-95 Δ*nheBC*Δ*sph* comP*sph* and 8, NVH 0075-95 Δ*nheBC*Δ*sph* comP*plc.*
(DOC)

Table S1 **Bacterial strains used in this study.**
(DOC)

Table S2 **Plasmids and oligonucleotides used in this study.**
(DOC)

Table S3 **Characteristics and toxin gene profiles of** ***B. cereus*** **strains used for cytotoxicity screening.**
(DOC)
