# Supplementary figures and images for: Concerted Action of Sphingomyelinase and Non-Hemolytic Enterotoxin in Pathogenic Bacillus cereus
Source: PLoS One. 2013 Apr 16;8(4):e61404. doi: 10.1371/journal.pone.0061404 (PMC3628865; doi:10.1371/journal.pone.0061404)

**Figure S1**

**
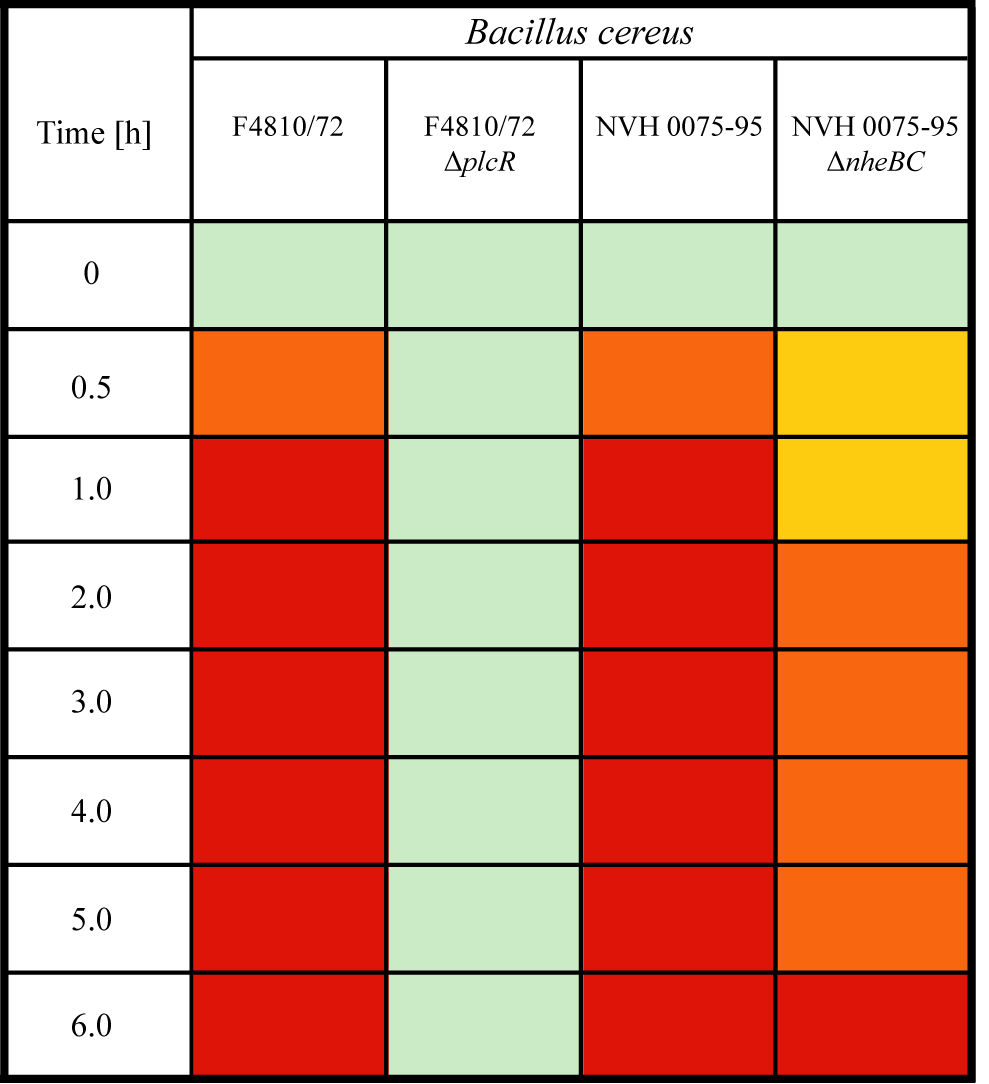
**

Supplement: Figure S1 — Cytotoxic effects of sterile B. cereus supernatants on IEC. Ptk6 cells were treated with B. cereus F4810/72 and NVH 0075-95 WT and isogenic mutant strains. Morphological changes of Ptk6 cells were monitored over time using light microscopy. All diluted supernatants (1∶2) caused immediate epithelial cell rounding and detachment except for the plcR deletion mutant. Intact monolayer (green), cell rounding <50% (yellow), cell rounding >50% (orange) and 100% cell detachment (red) are indicated. (DOC) [file pone.0061404.s001.doc]

**Figure S2**


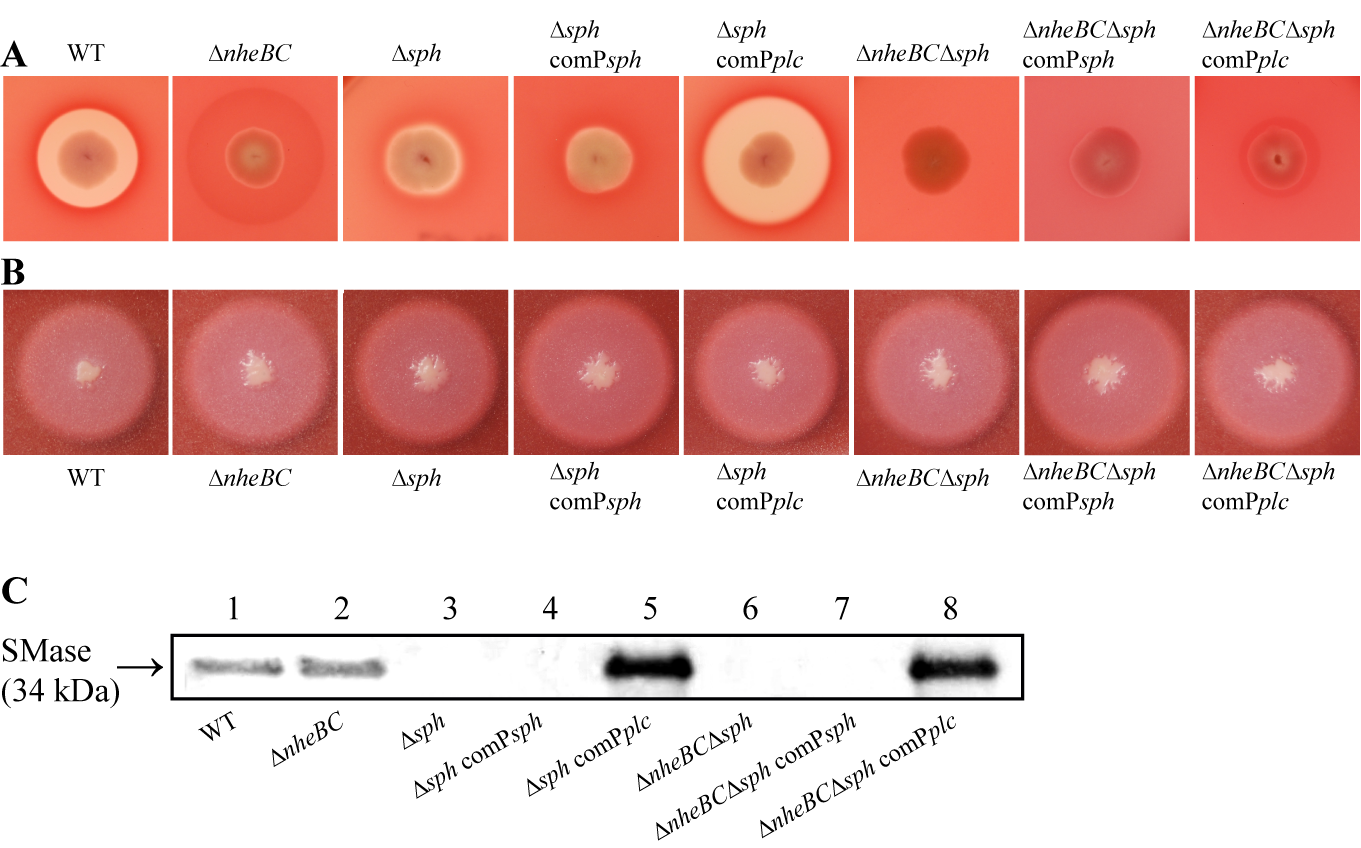

Supplement: Figure S2 — Characterization of sph deletion mutants, complemented and parental B. cereus strains. A. Hemolytic activity of B. cereus WT and isogenic mutant strains on Columbia agar (5% sheep blood, Oxoid). B. Colony morphology of WT and isogenic mutants on MYP (mannitol egg yolk polymyxin) agar indicating PC-PLC enzyme activity. C. Western blot analysis of SMase expression using a polyclonal anti-BcSMase antibody (1∶1000). Cells were grown in LB at 37°C and supernatants were harvested at similar OD600. Identical amounts (4 µg) of total protein preparations were separated on a 10% SDS-polyacrylamide gel and transferred to a PVDF membrane. Lanes: 1, B. cereus NVH 0075-95 (WT); 2, nheB truncation and nheC deletion mutant strain of NVH 0075-95 (ΔnheBC); 3, sph deletion mutant of NVH 0075-95 (Δsph); 4, NVH 0075-95 Δsph comPsph, sph deletion harboring pAD/sph/Psph/tet; 5, NVH 0075-95 Δsph comPplc, sph deletion complemented via pAD/sph/Pplc/tet driving sph transcription from the operon promoter region Pplc-sph; 6, nheBC inactivation and sph deletion mutant of NVH 0075-95 (ΔnheBCΔsph); 7, NVH 0075-95 ΔnheBCΔsph comPsph and 8, NVH 0075-95 ΔnheBCΔsph comPplc. (DOC) [file pone.0061404.s002.doc]

**Table S1.** Bacterial strains used in this study


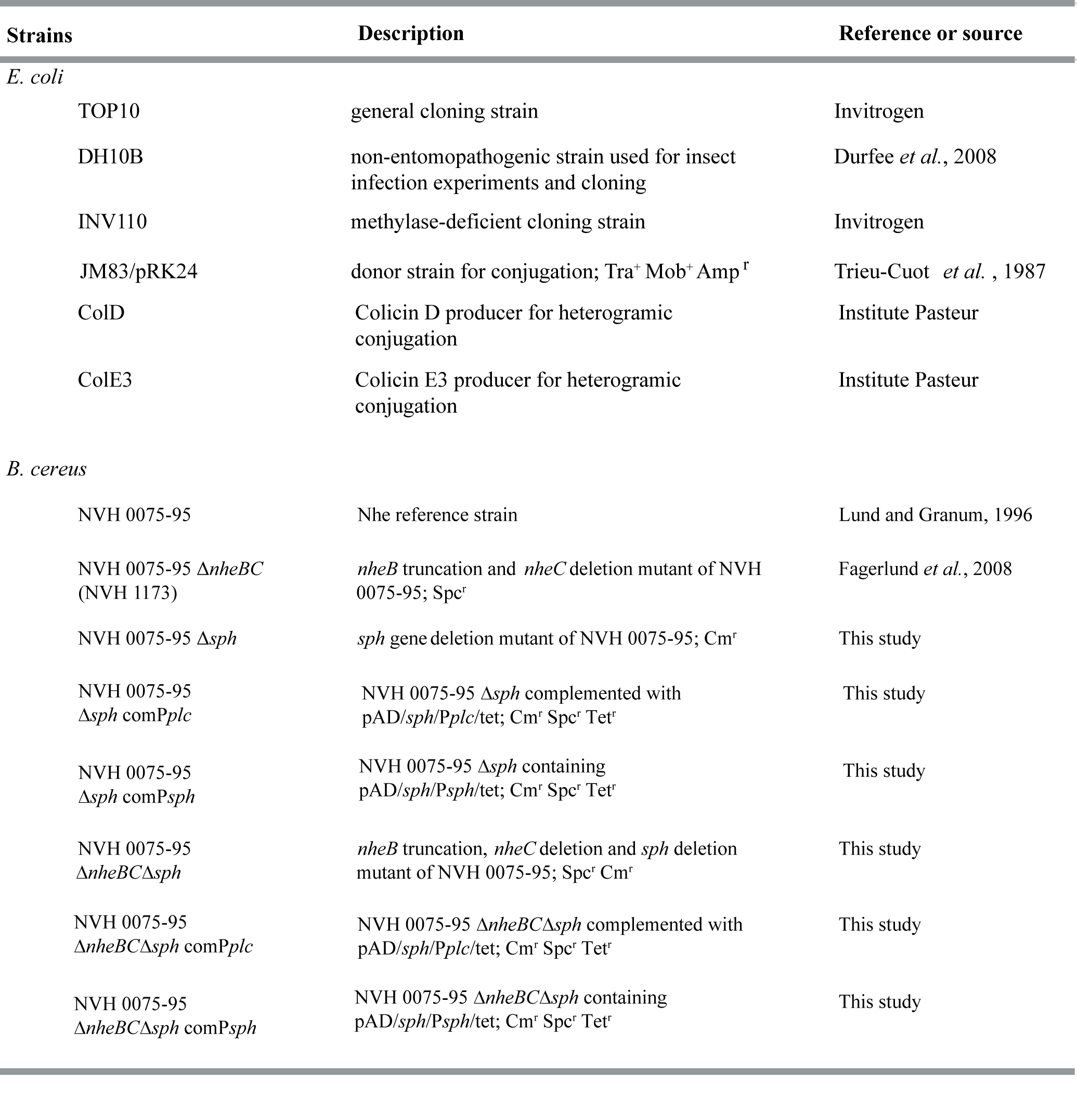

Supplement: Table S1 — Bacterial strains used in this study. (DOC) [file pone.0061404.s003.doc]

**Table S2.** Plasmids and oligonucleotides used in this study


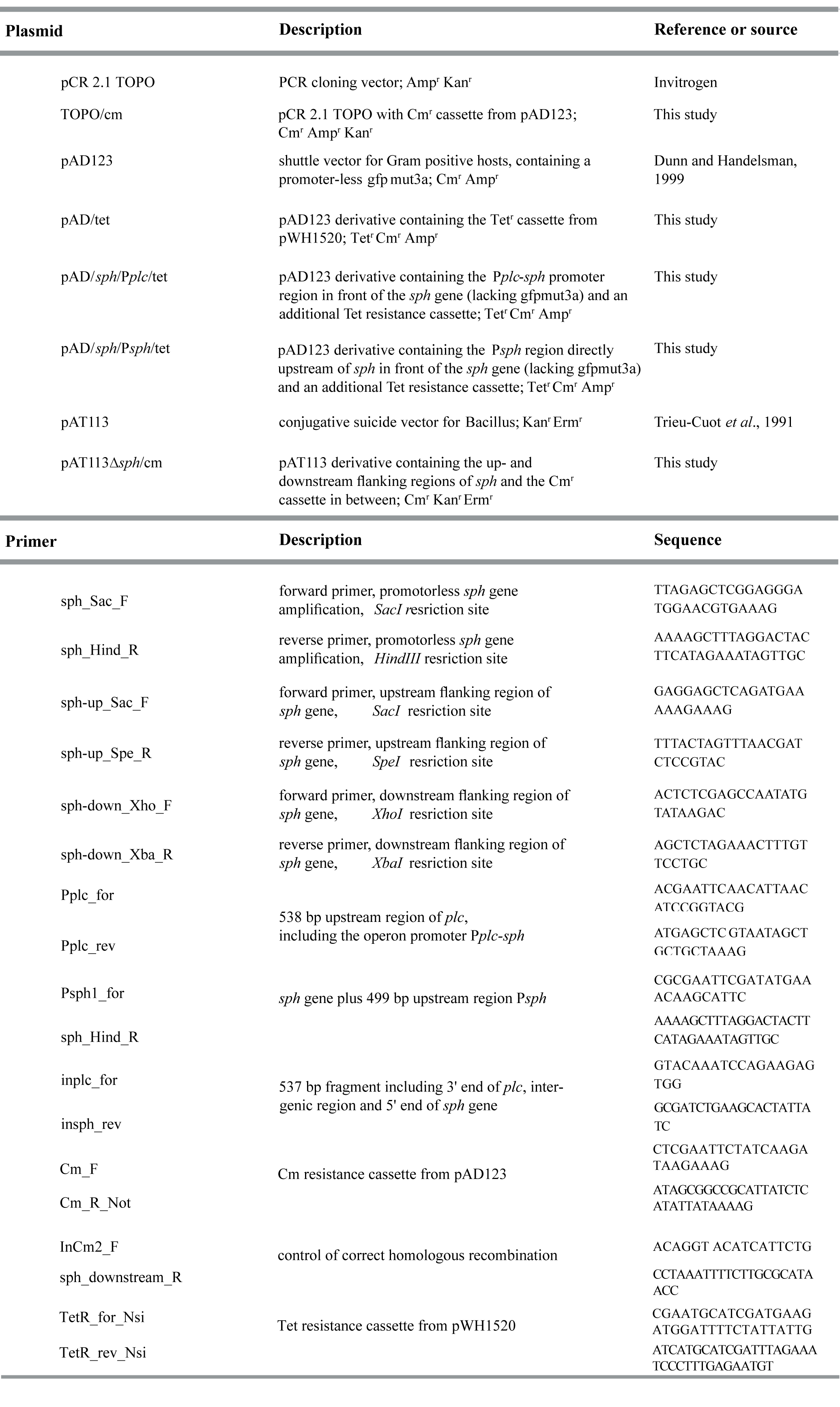

Supplement: Table S2 — Plasmids and oligonucleotides used in this study. (DOC) [file pone.0061404.s004.doc]
