# Supplementary material for: Concerted Action of Sphingomyelinase and Non-Hemolytic Enterotoxin in Pathogenic Bacillus cereus
Source: PLoS One. 2013 Apr 16;8(4):e61404. doi: 10.1371/journal.pone.0061404 (PMC3628865; doi:10.1371/journal.pone.0061404)
Supplement: Table S3 — Characteristics and toxin gene profiles of B. cereus strains used for cytotoxicity screening. (DOC) [file pone.0061404.s005.doc]

**Table S3.** Characteristics and toxin gene profiles of *B. cereus* strains used for cytotoxicity screening.


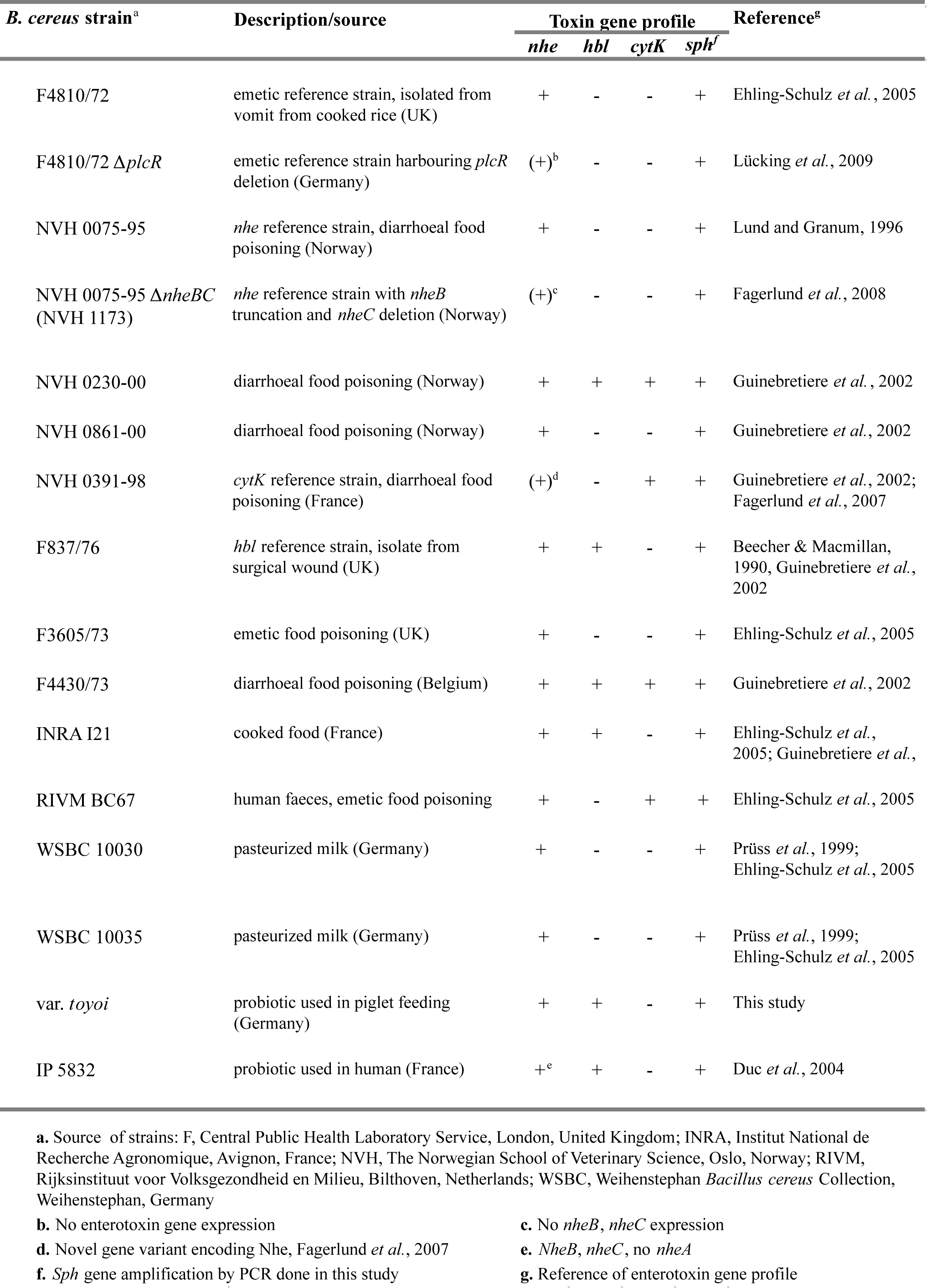


**Supplemental references**

Ehling-Schulz, M., Svensson, B., Guinebretiere, M. H., Lindback, T., Andersson, M., Schulz, A., Fricker, M., Christiansson, A., Granum, P. E., Martlbauer, E., Nguyen-The, C., Salkinoja-Salonen, M., and Scherer, S. (2005) Emetic toxin formation of *Bacillus cereus* is restricted to a single evolutionary lineage of closely related strains. *Microbiology* **151**, 183-197

Lücking, G., Dommel, M. K., Scherer, S., Fouet, A., and Ehling-Schulz, M. (2009) Cereulide

synthesis in emetic *Bacillus cereus* is controlled by the transition state regulator AbrB, but not by the

virulence regulator PlcR. *Microbiology* 155, 922-931

Lund, T., and Granum, P. E. (1996) Characterisation of a non-haemolytic enterotoxin complex from *Bacillus cereus* isolated after a foodborne outbreak. *FEMS Microbiol. Lett.* **141**, 151-156

Fagerlund, A., Lindbäck, T., Storset, A. K., Granum, P. E., and Hardy, S. P. (2008) *Bacillus cereus* Nhe is a pore-forming toxin with structural and functional properties similar to the ClyA (HlyE, SheA) family of haemolysins, able to induce osmotic lysis in epithelia. *Microbiology* **154**, 693-704

Guinebretière, M.H., Broussolle, V. and Nguyen-The, C. (2002) Enterotoxigenic profiles of food-poisoning and food-borne *Bacillus cereus* strains*.* *J. Clin. Microbiol.* **40**, 3053-3056

Fagerlund, A., Brillard, J., Fürst, R., Guinebretière, M.H. and Granum, P. (2007) Toxin production in a rare and genetically remote cluster of strains of the *Bacillus cereus* group*. BMC Microbiol.* **7** 43-50

Beecher, D.J. and Macmillan, J.D. (1990) A novel bicomponent hemolysin from *Bacillus cereus.* *Infect. Immun.* **58**, 2220-2227

Prüss, B.M., Dietrich, R., Nibler, B., Märtlbauer, E. and Scherer, S.(1999) The hemolytic enterotoxin HBL is broadly distributed among species of the *Bacillus cereus* group*.* *Appl. Environ. Microbiol.* **65**, 5436-5442

Duc, L., Hong, H. A., Barbosa, T. M., Henriques, A. O., and Cutting, S. M. (2004) Characterization of *Bacillus* probiotics available for human use. *Appl****.*** *Environ****.*** *Microbiol****.*** 70, 2161-2171

Dunn, A.K. and Handelsman, J. (1999) A vector for promoter trapping in *Bacillus cereus.* *Gene* **226**, 297-305

Trieu-Cuot, P., Carlier, C., Poyart-Salmeron, C. and Courvalinet, P. (1991) An integrative vector exploiting the transposition properties of Tn1545 for insertional mutagenesis and cloning of genes from gram-positive bacteria*.* *Gene* **106**, 21-27

Durfee, T., Nelson, R., Baldwin, S., Plunkett, G., Burland, V., Mau, B., Petrosino, J. F., Qin, X., Muzny, D. M., Ayele, M., Gibbs, R. A., Csörgo, B., Pósfai, G., Weinstock, G. M., and Blattneret, F. R. (2008) The Complete Genome Sequence of *Escherichia coli* DH10B: Insights into the Biology of a Laboratory Workhorse. *J. Bacteriol.* 190, 2597.

Trieu-Cuot, P., Carlier, C., Martin, P. and Courvalin, P. (1987) Plasmid transfer by conjugation from *Escherichia coli* to Gram-positive bacteria*.* *FEMS Microbiol. Lett.* **48,** 289-294
